# Antibody response and seroprevalence in healthcare workers after the BNT162b2 vaccination in a University Hospital at Tokyo

**DOI:** 10.1038/s41598-022-12809-x

**Published:** 2022-05-24

**Authors:** Gene Igawa, Tomohiko Ai, Takamasa Yamamoto, Kanami Ito, Shuko Nojiri, Kaori Saito, Mitsuru Wakita, Hiroshi Fukuda, Satoshi Hori, Shigeki Misawa, Takashi Miida, Kuniaki Seyama, Kazuhisa Takahashi, Yoko Tabe, Toshio Naito

**Affiliations:** 1 Department of Clinical Laboratory, Juntendo University Hospital, Tokyo, Japan; 2 Department of Clinical Laboratory Medicine, Juntendo University Faculty of Medicine, Hongo 2-1-2, Bunkyo-ku, Tokyo, 113-8421 Japan; 3 Department of Safety and Health Promotion, Juntendo University, Tokyo, Japan; 4 Medical Technology Innovation Center, Juntendo University, Tokyo, Japan; 5 Department of General Medicine, Juntendo University Graduate School of Medicine, Tokyo, Japan; 6 Department of Respiratory Medicine, Juntendo University Graduate School of Medicine, Tokyo, Japan; 7 Infection Control Unit, Juntendo University Hospital, Tokyo, Japan; 8 Department of Infection Control Science, Juntendo University Graduate School of Medicine, Tokyo, Japan; 9 Department of Research Support Utilizing Bioresource Bank, Juntendo University Graduate School of Medicine, Tokyo, Japan

**Keywords:** Virology, Microbiology, Health care, Medical research

## Abstract

In 2020, we reported a low seroprevalence of N-specific antibodies in 4147 health care workers (HCWs) at a frontline hospital in Tokyo, Japan. In Japan, a vaccine campaign was launched in early 2021. We re-evaluated seroprevalences of N- and S-specific antibodies in 2202 HCWs who took two doses of the BNT162b2 vaccine. In 2021, N-specific seroprevalence remains as low as 1.59%. The seroprevalences were comparable among all HCWs regardless of exposure levels. Almost all of the HCWs elicited S-specific antibodies after vaccination. However, the HCWs who had COVID-19 elicited higher S-specific antibody titers than those who did not have COVID-19. In the HCWs without a history of COVID-19, 1.1% (23 out of 2185) were seropositive with N-specific antibodies, indicating the existence of asymptomatic infections. Also, S-specific antibody titers were higher in females and younger HCWs, and in those who had severe side effects. However, S-specific antibody titers were lower depending on the number of days after the second dose of vaccination specifically in elderly individuals. In conclusion, this study indicates N-specific seroprevalence remains low in HCWs at a frontline hospital in Tokyo. The mRNA vaccine elicited S-specific antibody in HCWs, however, the titers decreased as the days proceeded.

## Introduction

The emergence of severe acute respiratory syndrome coronavirus 2 (SARS-CoV-2) in Wuhan China, the causative agent of coronavirus disease 2019 (COVID-19), led to the declaration of a pandemic by the World Health Organization (WHO) in 2020^1^. As a preventative measure, a worldwide vaccine campaign was launched. Several messenger RNA (mRNA) vaccines, such as BNT162b2 provided by Pfizer and BioNTech, were designed to produce the spike (S) proteins and generate S-specific antibodies in human lymphocytes. The preventive effects of BNT162b2 were up to 95% in phase 3 clinical trials^2^. Also, several clinical studies claimed the BNT162b2 vaccine may reduce disease severity^3–5^.

In the individuals infected with SARS-CoV-2, both Nucleocapsid (N)-specific antibodies and S-specific antibodies are expected to be produced whereas mRNA vaccines aim to generate only S-specific antibodies. Also, mRNA vaccinations showed booster effects even in individuals who previously had COVID-19^6,7^. Moreover, the S-specific antibodies may correlate with neutralizing activities examined using in vitro systems^8,9^. Therefore, various factors, such as sex and age, that can affect elicitation and decline of antibodies by vaccination need to be elucidated.

We previously reported, in the early phase of the pandemic, the seroprevalence of N-specific antibodies among healthcare workers (HCWs) was low and asymptomatic infection was rare despite significant exposure to SARS-CoV-2 in our hospital^10^. However, as the COVID-19 pandemic expanded, the seroprevalence of N-specific antibodies may have changed in HCWs who are at higher risk than the general population.

In the present study, we enrolled a large number of employees in our hospital who received the BNT162b2 mRNA vaccine. We sought to investigate the titers of S-specific antibodies in terms of sex, age, history of infection to SARS-CoV-2, and the side effects of the vaccination. We also evaluated the seroprevalence of N-specific antibodies in the HCWs between 2020 and 2021.

## Results

Characteristics of the participants who received the BNT162b2 vaccine are shown in Table 1. Few individuals (0.77%) were previously infected by SARS-CoV-2, diagnosed by real-time reverse transcriptase polymerase chain reaction (RT-PCR). The alpha variant was dominant in Japan during the study period^11^.

**Table 1:** Characteristics of healthcare workers in this study.

	Total	Male	Female
	2202	741	1461
**Age, *n* (%)**			
20–29	724 (32.9)	106 (4.8)	618 (28.1)
30–39	673 (30.6)	246 (11.2)	427 (19.4)
40–49	471 (21.4)	198 (9.0)	273 (12.4)
50–59	229 (10.4)	120 (5.4)	109 (5.0)
60–69	91 (4.1)	61 (2.8)	30 (1.4)
70–	14 (0.6)	10 (0.5)	4 (0.2)
**COVID-19 history, *n* (%)**			
Pre-vaccination	5 (0.23)	1 (0.13)	4 (0.27)
Post second vaccination	17 (0.77)	4 (0.54)	13 (0.89)
**Days from vaccination**			
Median (IQR)	62 (58–65)	63 (58–66)	62 (57–65)

**Figure 1:**
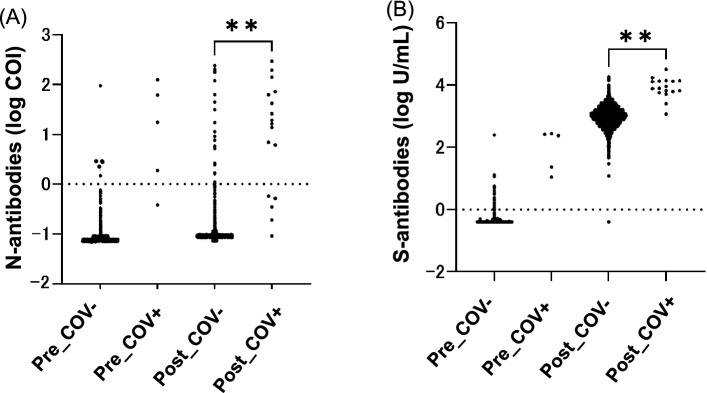
(**A**) Distribution of total N-specific antibodies in participants with and without history of COVID-19. Total N-specific antibody levels of pre-vaccination and post-vaccination participants with (COV+) and without (COV−) a history of COVID-19 were quantified. Statistical analysis was performed with t-testing between Post_COV- (n = 2185) and Post_COV+ (n = 17). **p value < 0.001. (**B**) Distribution of total S-specific antibodies in participants with and without history of COVID-19. Total S-specific antibody levels of pre-vaccination and post-vaccination participants with (COV+) and without (COV−) a history of COVID-19 were quantified. Statistical analysis was performed with t-testing between Post_COV- (n = 2185) and Post_COV+ (n = 17). **p value < 0.001.

Both the titers of N-specific and S-specific antibodies were higher in the group with a history of COVID-19 in both time points (Fig. 1A,B). Most individuals in the post second vaccination (post-vaccination) group elicited S-specific antibodies regardless of their history with COVID-19. In the pre-vaccination group all individuals with history of COVID-19 were seropositive with S-specific antibodies. However, 24 individuals were seropositive with S-specific antibodies in the group without a history of COVID-19, and only 1 out of the 24 was seropositive with N-specific antibodies.

Seropositive individuals with N-specific antibodies are considered as previously infected with SARS-CoV-2. Therefore, individuals with a history of COVID-19 were highly seropositive for N-specific antibodies: 80.0% (4/5) in the pre-vaccination group and 70.6% (12/17) in the post-vaccination group. All four N-specific seropositive individuals in 2020 showed lower titers in 2021 and one became seronegative (0.518 COI).

**Figure 2:**
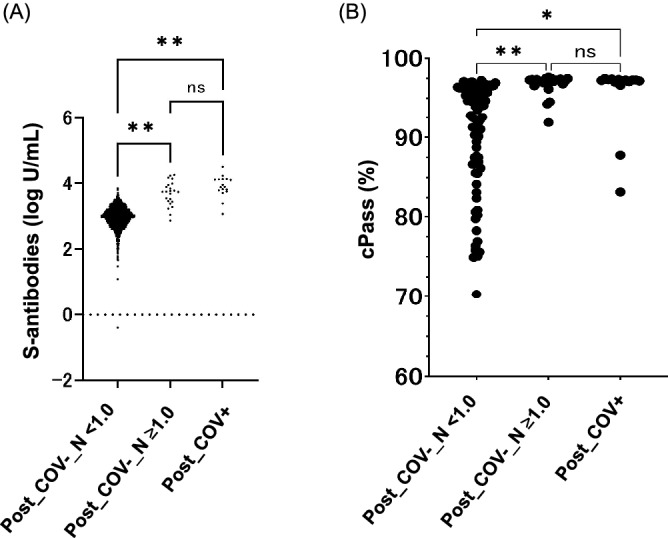
(**A**) Distribution of total S-specific antibodies in participants after second vaccination by N-specific antibody levels. Total S-specific antibody levels were quantified. Statistical analysis was performed with one-way ANOVA among N-negative post-vaccination participants without a COVID-19 history (Post_COV−_N < 1.0) (n = 2162), N-positive post-vaccination participants without a COVID-19 history (Post_COV−_N ≥ 1.0) (n = 23), and post-vaccination participants with a COVID-19 history (Post_COV+) (n = 17). **p value < 0.001. ns, no significant difference. (**B**) Distribution of cPass surrogate neutralizing antibody test values in participants after second vaccination by N-specific antibody levels. Surrogate neutralizing antibody values were quantified. Statistical analysis was performed with one-way ANOVA among N-negative post-vaccination participants without a COVID-19 history (Post_COV−_N < 1.0) (n = 86), N-positive post-vaccination participants without a COVID-19 history (Post_COV−_N ≥ 1.0) (n = 24), and post-vaccination participants with a COVID-19 history (Post_COV+) (n = 15). **p value < 0.001. *p value < 0.05. ns, no significant difference.

Five samples (0.23%; 5 out of 2198) in the pre-vaccination group and 23 samples (1.1%; 23 out of 2185) in the post-vaccination group without a history of COVID-19 were seropositive for N-specific antibodies (1.02 COI–239 COI). All five of these N-specific seropositive individuals in the pre-vaccination group retained seropositivity in 2021. Figure 2A shows levels of S-specific antibodies in various groups who took two doses of the vaccine. Compared to the N-specific antibody negative (COI < 1.0) and positive (COI ≥ 1.0) HCWs who were not previously diagnosed with COVID-19, S-specific antibody titers were statistically higher in HCWs who were positive for N-specific antibodies (COI ≥ 1.0) (p < 0.0001). The S-specific antibody titers of these N-positive HCWs were comparable with the HCWs who had been diagnosed with COVID-19.

Next, we examined the correlation between the S-specific antibody titers and the surrogate virus neutralizing antibody test (sVNT) results using a Genscript cPass assay (Genscript, Piscataway, New Jersey, USA). The results of 143 samples (15 with history and 128 without history of COVID-19) showed a very strong correlation between the two assays with a Spearman’s rho of 0.906 (Supplementary Fig. 1). Furthermore, the relationship between the sVNT and exposure to SARS-CoV-2 was also examined. Figure 2B shows that the sVNT results were higher in the HCWs who were positive for N-specific antibodies (COI ≥ 1.0) and who had confirmed COVID-19 compared to the individuals with negative N-specific antibody tests (COI < 1.0).

The seroprevalence of N-specific antibodies in different job categories are shown in Table 2. The seroprevalence was the highest in nurses and lowest in researchers. The seroprevalence was lower in HCWs who worked with COVID-19 outpatients and wards in all job groups. Nevertheless, there was no significant difference in seroprevalence among HCWs (HR 1.36%, MR 1.07%, LR 0.72%).

**Table 2:** Seroprevalence among healthcare workers according to the risk of exposure and professional category.

Participants	N Ab positive			
	Total		COVID-19 dedicated	
**High exposure risk (HR)**				
Medical doctors	(n = 671)	9 (1.34%)	(n = 242)	1 (0.41%)
Nurses	(n = 878)	16 (1.82%)	(n = 198)	5 (2.53%)
**Medium exposure risk (MR)**				
Laboratory personnel	(n = 179)	3 (1.68%)	(n = 5)	0 (0.00%)
Paramedics	(n = 198)	3 (1.52%)	(n = 15)	1 (6.67%)
**Low exposure risk (LR)**				
Administrative staff	(n = 244)	4 (1.64%)		
Researchers	(n = 23)	0 (0.00%)	(n = 5)	0 (0.00%)
Other	(n = 9)	0 (0.00%)		
All	(n = 2,202)	35 (1.59%)	(n = 465)	7 (1.51%)

**Figure 3:**
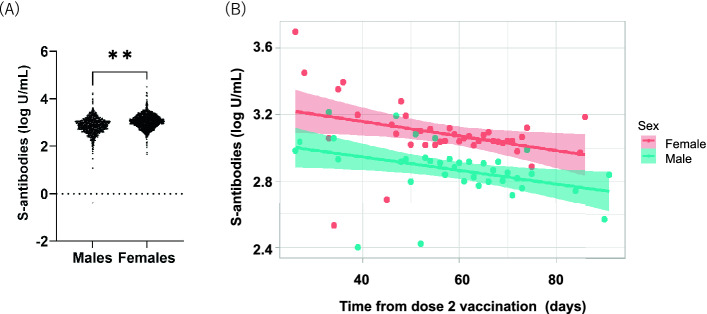
Distribution and reduction of total S-specific antibodies in participants after second vaccination by sex. (**A**) Total S-specific antibody levels were quantified. Statistical analysis was performed with student t testing between males (n = 741) and females (n = 1461). **p value < 0.001. (**B**) Scatterplot and regression line colors indicate antibody response for males and females. The 95% CIs are calculated by prediction ± 1.96 × standard error of prediction.

**Figure 4:**
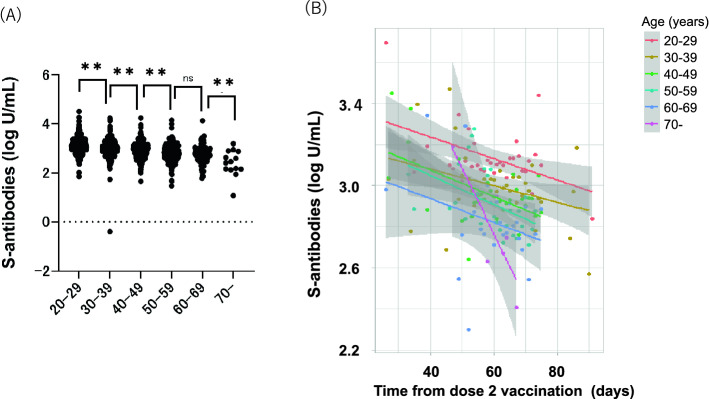
Distribution and reduction of total S-specific antibodies in participants after second vaccination among age group. (**A**) Total S-specific antibody levels were quantified. Statistical analysis was performed with one-way ANOVA among age 20–29 (n = 724), 30–39 (n = 673), 40–49 (n = 471), 50–59 (n = 229), 60–69 (n = 91), and 70 + (n = 14). **p value < 0.001. (**B**) Scatterplot and regression line colors indicate antibody response for the age groups mentioned in (**A**). The 95% CIs are calculated by prediction ± 1.96 × standard error of prediction. ns, no significant difference.

We also compared the S-specific antibody titers in terms of sex and age in the post-vaccination group. Females had higher titer of S-specific antibodies than males (*p* < 0.0001) (Fig. 3A). The median titer of S-specific antibodies was 1461 U/mL (IQR 757.5–1705) for females and 766 U/mL (IQR: 475.5–1178) for males. Regardless of sex, the S-specific antibody titers gradually decreased by time since their second vaccination (Fig. 3B). The titer of S-specific antibodies was higher in the younger age groups (Fig. 4A). The median titer of S-specific antibodies was 1290 U/mL in age 20–29, 1007 U/mL in age 30–39, 832 U/mL in age 40–49, 676.0 U/mL in age 50–59, 582 U/mL in age 60–69, and 272.5 U/mL in age 70+. Although the S-specific antibody titers gradually decreased in all age groups, participants of 70 years and over showed more prominent decline than younger participants (Fig. 4B).

Concurrently with the antibody response analysis, the side effects of vaccinations were also evaluated. Information regarding side effects was obtained from 2159 individuals after the second vaccination. Table 3 shows the proportion of the side effects and compared them with sex and their history of COVID-19. Females had major side effects more frequently than males, however, the proportion was low. Also, the type of side effects between sex were MORE compared and most of them were common in females with a significant difference (Supplementary Table 1). However, there was no significant difference in most symptoms between those with and without previous COVID-19 diagnosis except for malaise or anorexia (Supplementary Table 2).

**Table 3:** Side effects severity among sex and history of COVID-19.

	Minor side effects (N = 2047)	Major side effects (N = 112)	*p* value
**Sex, *n* (%)**			
Males	711(97.5%)	18 (2.5%)	< 0.0001
Females	1336 (93.4%)	94 (6.6%)	
**COVID-19 history, *n* (%)**			
Yes	17 (100%)	0 (0.0%)	> 0.9999
No	2030 (94.8%)	112 (5.2%)	

**Figure 5:**
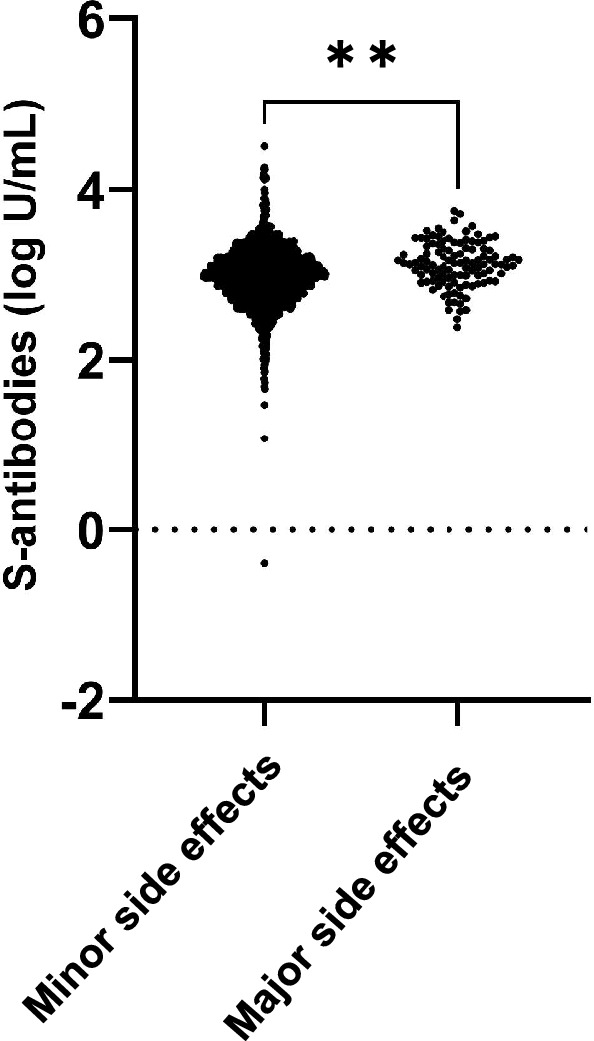
Distribution of total S-specific antibodies in participants after second depending on the side effects severity. Total S-specific antibody levels were quantified. Statistical analysis was performed with student t testing between minor side effects group (n = 2014) and major sides effect group (n = 112). **p value < 0.001.

Moreover, we evaluated the correlation between antibody titers and severity of side effects. Individuals with anaphylaxes as well as those who took more than two days off from work were considered as having major side effects, and those who took less than 1 day off from work were considered as having minor side effects. Eight individuals, all of whom had no history of COVID-19, had anaphylaxes. Females had major side effects more frequent than the male group (*p* < 0.0001). Median titer of S-specific antibodies was higher in the group with major side effects (1325 U/mL) than in the group with minor side effects (992.0 U/mL) (*p* < 0.0001) (Fig. 5).

## Discussion

This study was conducted in 2021 to evaluate the seroprevalence of N- and S-specific antibodies in a large number of HCWs at a first line hospital in Tokyo, Japan. In our previous study conducted in 2020, we reported the seroprevalence of N-specific antibodies was 0.34% in HCWs, and there was no significant difference among exposure levels to SARS-CoV-2^10^. Though the participants in the current study were not exactly the same as the participants in our previous study, the N-specific seroprevalence slightly increased but still remains low (1.59%). Similarly, several studies from Asian countries reported that the seroprevalences were low (0%-3.8%) in HCWs^12,13^. Conversely, studies from European countries and the U.S. reported that HCWs have high seroprevalences (11%-31.6%)^14–16^. However, these studies were conducted in 2020. Therefore, as a result of the expanding COVID-19 pandemic, the gap in seroprevalence may be larger at this time.

Though the specificity of the Elecsys^®^ Anti-SARS-CoV-2 S test is 99.98%, we identified 23 individuals who showed positive results for the S-specific antibody, but not the N-specific antibody, without history of COVID-19. It has been reported that the titer of N-specific antibodies decline more rapidly over time compared to the S-specific antibodies, especially in mild-moderate patients^17,18^. Indeed, the titers of N-specific antibodies in five individuals who had confirmed COVID-19 in 2020 decreased in 2021. Therefore, these 23 individuals might have had an asymptomatic infection although a false positive result for the S-specific antibody cannot be completely excluded.

As shown in Fig. 2, high titer of S-specific antibodies was observed in N-specific seropositive individuals who have not been diagnosed as COVID-19 by RT-PCR since they had no COVID-19 related symptoms. S-specific antibody titers of N-positive individuals were comparable to those of COVID-19 infected cases. Although the overall seroprevalences of HCWs increased in 2021, the seroprevalence in the COVID-19 dedicated HCWs was still low. Furthermore, there was no significant difference between job categories grouped by exposure risk to SARS-CoV-2, which is inconsistent with previous studies indicating high seroprevalence in HCWs exposed to COVID-19^14,15^. This inconsistency may be due to the lower prevalence of COVID-19 and less contact with SARS-CoV-2 in Japan.

Currently, the neutralizing activity of the detected S-specific antibodies after vaccination is one of the concerns. Therefore, Genscript cPass SARS-CoV-2 Antibody Detection Kit, a sVNT, was developed and correlated well with the plaque reduction neutralizing test (PRNT) that is considered a gold standard to assess the neutralizing proteins^19,20^. Also, concordant with our results, S-specific antibody titers correlated well with the sVNT^21–23^. Moreover, FDA claims 132 U/mL is required for convalescent plasma with high titers^24^. According to these criteria, 98.6% (2160 out of 2185) of individuals without a history of COVID-19 and all 17 individuals with a history of diagnosed COVID-19 obtained a sufficiently high amount of neutralizing antibodies. Concordant with the previous reports^25–27^, our results showed females and younger participants had higher S-specific antibody titers with a slow decline by time since second vaccination. The decline was more prominent in older than younger participants.

Our data shows that females had side effects more frequently than males. Side effects may be due to the polyethylene glycol (PEG) in the nanoparticles in mRNA vaccines^28^. Although PEG is considered safe, PEG related anaphylaxes have been reported^28^. Also, anaphylaxes after vaccination occur more frequently in females than males^29^. We observed that individuals who exhibited serious side effects had higher S-specific antibody titers, concordant with the previous report^26^. However, the information gathered by questionnaire is subjective. In fact, CDC reported 61 (34.9%) of 175 allergic cases were considered nonallergic after the case review^30^. Thus, the correlation between severity of side effects and S-specific antibody titers still remain unclear and further investigation is needed.

HCWs are on the front line facing COVID-19. Although using appropriate PPE including face guard and N95 masking, the risk of exposure to SARS-CoV-2 is high for HCWs. A study from Kobe City, located 350 miles away from Tokyo, reported that the seroprevalences of N-specific antibodies in individuals who had regular checkups increased from 0.4% in October 2020 to 2.1% in August 2021^31^. The overall seroprevalence of N-specific antibodies in our study was 1.59% which was comparable with the seroprevalence in Kobe City. This may indicate that the appropriate use of PPE is well conducted in our hospital.

This study has several limitations: (1) the present study was conducted in a single university hospital; (2) participants were HCWs who are relatively healthy and predominantly younger, and approximately 75% of the two youngest groups combined (20–30 s) were women; (3) chronological changes in antibody titers could not be followed up in the same individuals; and (4) the authentic neutralizing antibody activities could not be evaluated.

In conclusion, the seroprevalence of N-specific antibodies of HCWs remain low in this frontline hospital in Tokyo, Japan. Though the BNT162b2 mRNA vaccine elicited detectable S-specific antibodies in our cohort, the titers decreased overtime. Therefore, further investigation using samples at multiple time points are warranted to elucidate the role of vaccination in protecting HCWs.

## Materials and methods

### Study participants

In total, 2202 people participated in the study at Juntendo University. The serum samples of participants who have received the BNT162b2 vaccine by May 13, 2021 at the university were collected from the residual blood samples taken at the annual health checkups (June 2021) at the university. Serum collected in 2020, which were stored in − 80 ℃, were used as pre-vaccination samples. Side effects information of the vaccination was obtained by questionnaire after the second dose.

The research related to human use has complied with all the relevant national regulations and institutional policies and was conducted in accordance with the tenets of the Helsinki Declaration. It has been approved by the Institutional Review Board (IRB) at Juntendo University (IRB # M20-0089-M01). Informed consents were obtained from all individuals included in this study.

### Serological testing

The antibodies against SARS-CoV-2 were measured using Elecsys^®^ Anti-SARS-CoV-2 (Roche Diagnosis, Basel, Switzerland) and Elecsys^®^ Anti-SARS-CoV-2 S (Roche Diagnosis, Basel, Switzerland) on cobas e 801 analyzer according to the manufacturer’s instructions. Elecsys^®^ Anti-SARS-CoV-2 detects the N-specific total immunoglobulin, and the results are presented in the form of a cutoff index (COI; signal sample/cutoff) with qualitative results. COI ≥ 1.0 was interpreted as positive. Elecsys^®^ Anti-SARS-CoV-2 S detects the specific total antibodies against the receptor binding domain (S-RBD) of the S antigen, and the results are presented in the form of U/mL with qualitative results. Greater than or equal to 0.8 U/mL is interpreted as positive, and the values over the measurement range (250 U/mL) were quantified with dilution by the recommended Diluent Universal on the cobas e 801 analyzer.

### SARS-CoV-2 neutralizing test

Neutralizing antibodies were measured by GenScript cPass^®^ SARS-CoV-2 Antibody Detection Kit, a blocking enzyme-linked immunosorbent assay (GenScript, Piscataway, New Jersey, USA), following the company’s instructions. Briefly, the samples and controls were pre-incubated with the HRP-labeled recombinant RBD proteins, then, the mixture was added to the capture plate pre-coated with the hACE2 proteins. After the complex of neutralizing antibody with RBD-HRP was removed by washing, the wells were read at 450 nm in a microtiter plate reader. The percent signal inhibition for the detection of neutralizing antibodies were calculated as follows:

$$ \begin{aligned} \% {\text{ Signal Inhibition}} & = ({1} - {\text{OD value of Sample}}/{\text{OD value of Negative Control}}) \\ & \quad \times {1}00\% \, \left( {{\text{cutoff value: 3}}0\% {\text{ signal inhibition}}} \right). \\ \end{aligned} $$

### Statistical analysis

Statistical analysis was performed on GraphPad Prism version 9.2.0 for Windows (GraphPad Software, San Diego, California, USA, www.graphpad.com). Qualitative antibody levels were compared with Student t testing and one-way ANOVA followed by Bonferroni multiple comparison. Fisher’s exact testing was used to evaluate significant differences in ratio among groups. Correlation between the surrogate neutralizing antibody test and S-specific antibody titers was evaluated by Spearman’s rank-order correlation coefficient (rho). A two-tailed *p* value of < 0.05 was considered statistically significant. The regression model analysis was conducted between S-specific antibodies and days from second vaccination. For interaction between age group and days from vaccination regarding antibody level, ANOVA analysis with interaction term analysis was performed after removing abnormal values. The model analysis was performed using R statistical software version 4.1.0.

## Supplementary Information

Supplementary Figure S1.

Supplementary Tables.
