# Supplementary material for: Antibody response and seroprevalence in healthcare workers after the BNT162b2 vaccination in a University Hospital at Tokyo
Source: Sci Rep. 2022 May 24;12:8707. doi: 10.1038/s41598-022-12809-x (PMC9127282; doi:10.1038/s41598-022-12809-x)
Supplement: Supplementary file 1 — Supplementary Figure S1. [file 41598_2022_12809_MOESM1_ESM.pdf]

Supplemental Information

Antibody Response and Seroprevalence in Healthcare Workers after the BNT162b2 Vaccination in a University Hospital at Tokyo

Gene Igawa[1], Tomohiko Ai[2], Takamasa Yamamoto[1], Kanami Ito[3], Shuko Nojiri[4], Kaori Saito[2], Mitsuru Wakita[1], Hiroshi Fukuda[3,5], Satoshi Hori[6,7,8], Shigeki Misawa[1], Takashi Miida[2], Kuniaki Seyama[3,5], Kazuhisa Takahashi[6,9], Yoko Tabe[2,9*], Toshio Naito[3,5,9]

1; Department of Clinical Laboratory, Juntendo University Hospital, Tokyo, Japan.

2; Department of Clinical Laboratory Medicine, Juntendo University Faculty of Medicine, Tokyo, Japan.

3; Department of Safety and Health Promotion, Juntendo University, Tokyo, Japan.

4; Medical Technology Innovation Center, Juntendo University, Tokyo, Japan.

5; Department of General Medicine, Juntendo University Graduate School of Medicine, Tokyo, Japan.

6; Department of Respiratory Medicine, Juntendo University Graduate School of Medicine, Tokyo, Japan.

7; Infection Control Unit, Juntendo University Hospital, Tokyo, Japan.

8; Department of Infection Control Science, Juntendo University Graduate School of Medicine, Tokyo, Japan.

9; Department of Research Support Utilizing Bioresource Bank, Juntendo University Graduate School of Medicine, Tokyo, Japan.

*Corresponding author

Yoko Tabe

E-mail: tabe@juntendo.ac.jp

Full postal address: Hongo 2-1-2, Bunkyo-ku, Tokyo 113-8421, Japan.

Supplemental Figure

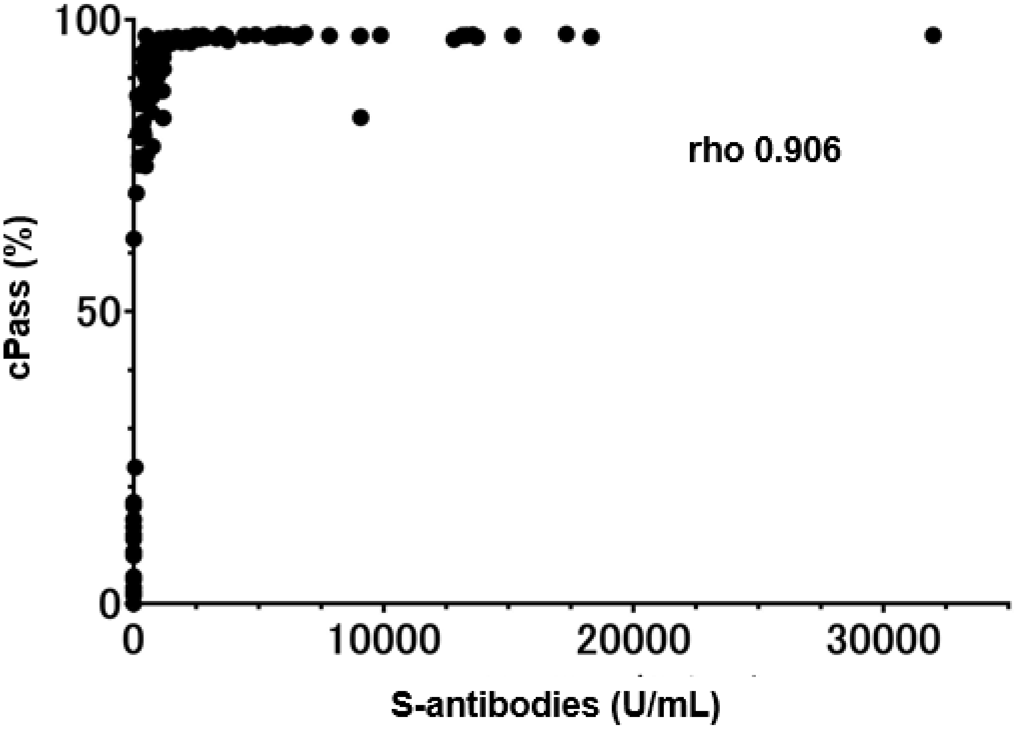

Figure S1. Comparison of cPass surrogate neutralizing antibody test values and S-specific antibody titers. Correlation of the cPass surrogate neutralizing antibody test values (%) and S-specific antibody titers (U/mL) was evaluated by Spearman's rank-order correlation coefficient (rho).
